# A clinical study on medication overuse headache in childhood and adolescence

**DOI:** 10.1186/1129-2377-14-S1-P27

**Published:** 2013-02-21

**Authors:** F Piazza, M Chiappedi, E Maffioletti, F Galli, G Spada, G Nappi, U Balottin

**Affiliations:** 1 Child Neuropsychiatry Unit – IRCCS “C. Mondino” (Pavia), Italy; 2 Don Carlo Gnocchi ONLUS Foundation – Milan, Italy; 3 Child Neuropsychiatry, University of Pavia, Italy; 4 Headache Science Centre of the IRCCS ‘National Institute of Neurology C. Mondino’ Foundation, Pavia, Italy

## Introduction

There are few studies on Medication Overuse Headache (MOH) in children and no epidemiological studies on an Italian population. We evaluated the prevalence of MOH in patients referred to our Center in 2011.

## Methods

We studied 118 patients looking for correlations between age of onset, sex, age at first contact, headache type, presence of Chronic Daily Headache (CDH), pain frequency, severity and presence of MOH (according to the revised criteria [1]) by Student’s t test, Pearson’s Chi Square and Mann-Whitney’s test were used to analyze data.

## Results

44.9% of the sample had a diagnosis of CDH. Among this group the prevalence of medication overuse was 20.8%. After 2 months of drug withdrawal 45.4% of the patients reported a significant improvement. No significant correlation was found between the presence of MOH and age of onset, sex, age at first contact, headache type, pain frequency and severity.

## Conclusion

About 1/5 of children with CDH afferent to our Headache Unit are at high risk of worsening because of medication overuse. No previous studies showed a role for medication overuse in the etiopathogenesis of CDH in children and adolescents [2]. We suggest to keep in mind the possibility of medication overuse in children with headache and to investigate it carefully. Further studies are required to define a specific treatment protocol.
